# Asleep motor mapping in resected low-grade gliomas -a population based multicenter study

**DOI:** 10.1016/j.bas.2025.105918

**Published:** 2025-12-23

**Authors:** Sophia M. Leiss, Margret Jensdottir, Ole Solheim, Alba Corell, Anna Lipatnikova, Sasha Gulati, Klas Holmgren, Francesco Latini, Ruby Mahesparan, Peter Milos, Alice Neimantaite, Henrietta Nittby Redebrandt, Lars Kjelsberg Pedersen, Rickard L. Sjöberg, Björn Sjögren, Gregor Tomasevic, Erik Thurin, Maria Zetterling, Jiri Bartek, Asgeir S. Jakola

**Affiliations:** a Institute of Neuroscience and Physiology, Sahlgrenska Academy, University of Gothenburg, Gothenburg, Sweden; b Department of Clinical Neuroscience, Karolinska Institutet, Stockholm, Sweden; c Department of Neurosurgery, Karolinska University Hospital, Stockholm, Sweden; d Department of Neuromedicine and Movement Science, Faculty of Medicine and Health Sciences, Norwegian University of Science and Technology, NTNU, Trondheim, Norway; e Department of Neurosurgery, St. Olavs Hospital, Trondheim University Hospital, Trondheim, Norway; f Department of Neurosurgery, Sahlgrenska University Hospital, Gothenburg, Sweden; g Department of Medical Sciences, Section of Neurosurgery, Uppsala University Hospital, Uppsala, Sweden; h Department of Clinical Sciences, Neuroscience, Umeå University, Umeå, Sweden; i Department of Neurosurgery, University Hospital of Northern Sweden, Umeå, Sweden; j Department of Clinical Medicine, Faculty of Medicine, University of Bergen, Bergen, Norway; k Department of Neurosurgery, Haukeland University Hospital, Bergen, Norway; l Department of Neurosurgery, Linköping University Hospital, Sweden; m Department of Biomedical and clinical Sciences, Linköping University, Linköping, Sweden; n Department of Clinical Sciences, Lund University, Lund, Sweden; o Department of Neurosurgery, Skåne University Hospital, Lund, Sweden; p Department of Neurosurgery, University Hospital of North Norway, Tromsø, Norway; q Department of Radiology, Sahlgrenska University Hospital, Gothenburg, Sweden; r Department of Neurosurgery, Copenhagen University Hospital Rigshospitalet, Copenhagen, Denmark

**Keywords:** Asleep mapping, Motor deficits, Low grade glioma

## Abstract

**Introduction:**

Maximal safe resection is established goal of WHO grade 2 low-grade gliomas (LGG). Asleep motor mapping offers an alternative to awake surgery for tumors near motor areas and has been shown to be safe and effective in expert centers.

**Research question:**

We aimed to identify predictors of postoperative motor deficits, and describe patient selection and intraoperative mapping techniques across Scandinavian centers.

**Material and methods:**

We retrospectively analyzed patients (≥18) with WHO grade 2 gliomas who underwent asleep motor mapping across multiple Scandinavian neurosurgical centers. Clinical, surgical, and imaging data were extracted from medical records. The primary outcome was registered permanent postoperative motor deficits at 3 months. Associations with pre-, intraoperative, and radiological variables - including diffusion-weighted imaging (DWI) changes - were assessed using univariate and multivariate logistic regression.

**Results:**

We included 74 patients from eight institutions. Median age was 48 years, 38 (51.4 %) were female and median preoperative tumor volume was 43.2 ml. 13 (17.6 %) patients achieved gross-total resection and median postoperative volume was 7.8 ml. Permanent postoperative motor deficits occurred in 19 cases (25.7 %), and 5 (6.8 %) were considered major deficits. In univariate analysis, preoperative motor deficits (p = 0.009), postoperative DWI changes (p = 0.022), and age (p = 0.043) were significantly associated with new or worsened permanent deficits. Only DWI changes and age was confirmed in penalized multivariate logistic regression.

**Discussion and conclusion:**

Postoperative motor deficits were common despite use of asleep motor mapping. Preoperative motor deficits and diffusion-weighted imaging changes are predictors of permanent motor deficits in this setting.

## Highlights

• One in four patients had permanent motor deficits after surgery.
• Diffusion-weighted imaging changes predicted permanent motor deficits.
• Older age predicted permanent motor deficits.
• Pre-surgery motor problems linked in simple tests, not confirmed in full model.

## 1 Introduction

Surgical resection remains the cornerstone of treatment for diffuse low-grade gliomas (LGG). Optimizing motor outcome is important as motor deficits negatively impact quality of life and are associated with decreased survival (Correia et al., 2021; Sant et al., 2012). To minimize risks in motor eloquent regions, asleep motor mapping is a validated alternative to awake craniotomy (Lemaitre et al., 2022; Freyschlag and Duffau, 2014; Duffau, 2020; Jahangiri et al., 2020). Asleep motor mapping is not a single, standardized technique, but typically combines monitoring modalities such as transcranial motor evoked potentials (tcMEP) and/or continuous cortical MEP monitoring via strip electrodes, and direct cortical and subcortical mapping. A recent survey among neurosurgeons confirmed the variability and diversity in protocols and adjuncts used for motor mapping in clinical practice (Mansouri et al., 2023).

Asleep motor mapping is particularly valuable when awake surgery is not feasible due to anatomical, neurological, or patient-specific factors. It is frequently used for tumors in the primary motor cortex or supplementary motor area or in tumors in proximity with the corticospinal tract (Al-Adli et al., 2023). An asleep motor mapping protocol that has gained popularity is the Taniguchi method, using high-frequency stimulation that may hold lower seizure risk compared to the more traditional Penfield method (Jahangiri et al., 2022). Subcortical stimulation thresholds offer guidance on proximity to the corticospinal tract and offers resection guidance while intact MEPs is critical to evaluate the integrity of the entire system (Seidel et al., 2013). Recent studies support the safety of asleep motor mapping, reporting low rates of permanent motor deficits in expert centers (Morshed et al., 2024; Gogos et al., 2021). Identified risk factors for deficits include insular tumor location and preoperative motor deficits.

A study by Munkvold et al. highlighted substantial heterogeneity in the diagnostic work-up and surgical strategies for LGG across centers, underscoring the lack of standardization even within similar healthcare systems (Munkvold et al., 2021). Further, there is a lack of reports from population-based studies across institutions to better understand case and technique selection, and postoperative outcomes. These factors point to the need for data to evaluate use and outcomes of asleep motor mapping in a broad population-based multicenter setting.

The primary objective of this multicenter study was to identify predictors of postoperative motor deficits after asleep motor mapping for LGG. The secondary objectives were to describe patient selection, intraoperative mapping and monitoring techniques across Scandinavian centers.

## 2 Materials and methods

### 2.1 Patient population

This retrospective study is based on a Scandinavian multicenter collaboration (STAR cohort) involving nine neurosurgical departments in Sweden and Norway. It includes adult patients (≥18 years) with histopathological confirmed supratentorial WHO grade 2 gliomas, who underwent primary surgery between 2012 and 2017. In this study, the classification system used were those present at the time of treatment, and tissue were not systematically reclassified (Louis et al., 2007, 2016). For this sub-study, we identified patients who underwent asleep motor mapping (center-level utilization in Suppl. Table 1) with documented motor indications. Clinical, radiological, and surgical data were retrieved retrospectively from the local hospital PACS (Picture Archiving and Communication System, Sectra™) and from medical records.

### 2.2 Variables

At baseline, clinical presentation and performance status were recorded. Intraoperative variables captured details of the mapping approach (bipolar vs. monopolar stimulation, cortical and subcortical mapping), and occurrence of intraoperative seizure. Surgical decision-making variables included reasons for stopping resection (e.g., positive stimulation, perceived gross-total resection). Notably, routine IDH testing was not uniformly implemented across centers in the study period (2012–2017).

The presence of new or worsened postoperative deficits was recorded. Resolution or persistence of deficits was determined based on clinical neurological examinations documented in the patient records at discharge and follow-up visits. Deficits were classified as transient if resolved within three months, and as permanent otherwise. Permanent deficits were further categorized as minor or major based on their severity of paresis. This classification follows a functional-impact definition: *minor* deficits had limited impact on daily life, such as the ability to work, drive, travel, or perform physical activities. *Major* deficits, in contrast, led to significant limitations in these areas, impairing the ability to live a normal socio-professional life. This classification corresponds to the MRC (Medical Research Council) muscle power scale, where a grade of 4 is considered minor and grade 3 and lower is considered major, as reported by De Witt Hamer (De et al., 2012) seen in Suppl. Table 2. SMA syndromes (pure motor and motor–verbal, with transient or permanent deficits noted, respectively) were recorded separately. Only permanent pure-motor SMA cases were included in the analysis of permanent motor deficits, whereas transient and motor-verbal SMA cases were analyzed descriptively. Postoperative complications within 30 days were graded using the Landriel Ibanez classification (Landriel Ibañez et al., 2011), which categorizes adverse events by severity and required treatment. Grade I include non–life-threatening complications not requiring invasive procedures, Grade II involves complications needing surgical or endoscopic intervention, Grade III includes life-threatening events requiring ICU care, and Grade IV represents death. Complications were further classified as surgical (procedure-related) or medical (systemic), and in patients with multiple events, the most severe complication was recorded. Neurological deficits were reported separately and not included in the general complication count.

### 2.3 Assessment of ischemic lesions

Postoperative motor deficits may also result from iatrogenic injury unrelated to direct resection of eloquent brain regions. Ischemic lesions visible on early postoperative diffusion-weighted imaging (DWI) are one such mechanism (Zetterling et al., 2020; Berger et al., 2021; Tsuchiya et al., 2024; Dunås et al., 2023). In this dataset, eloquence was defined according to the UCSF scoring system which serves as a long-term prognostication for hemispheric LGG and is made up of areas of eloquence, patient age >50 years, KPS score ≤80, and lesion diameter >4 cm) (Chang et al., 2008). When lesions are near eloquent areas even small ischemic areas can be relevant (Strand et al., 2021). To better understand the usefulness of the technique in preventing permanent motor deficits, both the direct damage and indirect damage via ischemia must be considered. Radiological evaluations for ischemia were included if their DWI imaging was of sufficient quality and included both ADC and b1000 sequences. Areas of ischemia were identified as regions showing high signal intensity on b1000 images along with low ADC values, provided the signal exceeded 3 mm in thickness and was not attributable to postoperative hemorrhage on T1-weighted imaging. These areas were segmented volumetrically in 3D Slicer (version 5.6.2) by a trained evaluator (AL), in consultation with a radiologist (ET). Based on morphology, lesions were categorized as rim, sector or combination types. Significant DWI changes were identified as regions showing high signal intensity on b1000 images along with low ADC values, provided the signal exceeded 3 mm in thickness and was not attributable to postoperative hemorrhage on T1-weighted imaging.

### 2.4 Heatmap of spatial tumor distribution

To assess the location distribution of the tumors resected using asleep-motor mapping, a tumor localization heatmap was created. Tumor segmentation and registration of all tumor segmentations to a common space, MNI-space (Fonov et al., 2009), was applied as previously described (Gómez et al., 2021). A visual control of the registration results was done, and registration errors were adjusted with re-registration or landmark registration. The registered tumor segmentations were overlaid and a colormap was applied to create a tumor location heatmap. A ventricle mask was applied on the tumor overlay. Additionally, precentral gyrus from the Cerebrum Atlas was added on top of the tumor location heatmap to ease the interpretation of tumor locations (Manera et al., 2020). The 3D Slicer software (Fedorov et al., 2012) and Python programming language were utilized. ChatGPT was used in programming assistance to create the images of the brain in sagittal plane with lines showing the chosen slices of the heatmap.

### 2.5 Statistics

Initial analyses were conducted in SPSS (v30) to explore associations with any postoperative motor deficits. Due to the high incidence of transient deficits, subsequent analyses focused on permanent deficits, using Python (pandas (McKinney, 2011), scipy (Virtanen et al., 2020), statsmodels (Seabold and Perktold, 2010)) via Jupyter Notebook V4.2.5. Descriptive statistics summarized patient characteristics, lesion types, and ischemic lesion volumes. Associations were assessed using chi-square, ANOVA, or Fisher's exact test. The following variables were included: “Language deficit”, “Motor deficit”, “Asymptomatic, MRI due to unrelated issues”, “Clinical deterioration before surgery”, “Karnofsky performance status”, “Initial ‘wait-and-scan'", “Demonstrated tumor growth prior to surgery”, “MR spectroscopy”, “Amino acid PET”, “Main tumor location”, “Multifocal”, “Contrast enhancement”, “Laterality”, “Significant DWI changes”, “Histopathology higher grade”, “MRI new nodular contrast enhanced lesion >1 cm”, “MRI new ring-like glioblastoma pattern”, “1p19q status”, “Molecular data, IDH status”, “nTMS preoperatively”, “DTI preoperatively”, and “Tumor size at latest exam prior to surgery, volumetric (performed centrally).”

Variables with p < 0.5 in univariate analysis were considered for multivariate logistic regression. Preoperative motor deficits, significant DWI changes, and age were included in the final model. To reduce overfitting and address multicollinearity, penalized logistic regression with L2 (ridge) penalty was used. We compared stimulation modality groups (“combined cortical and subcortical mapping” versus “single-method mapping” - either cortical or subcortical alone) using chi-square tests for outcomes including motor deficits (any and permanent), intra- and postoperative seizures/status epilepticus, and complication severity (Ibañez classification grade ≥2). Intraoperative seizures were further analyzed in relation to stimulation parameters (mode, intensity), seizure prophylaxis, and mapping technique, using chi-square and Mann–Whitney U tests.

### Ethical approval

Ethical approval was granted by the Swedish Ethical Review Authority (EPN reference 705/17) and the Regional Committee for Medical and Health Research Ethics in Central Norway (REC reference 2017/1780). The need for informed consent was waived by the committees.

## 3 Results

### 3.1 Preoperative characteristics

**Fig. 1:**
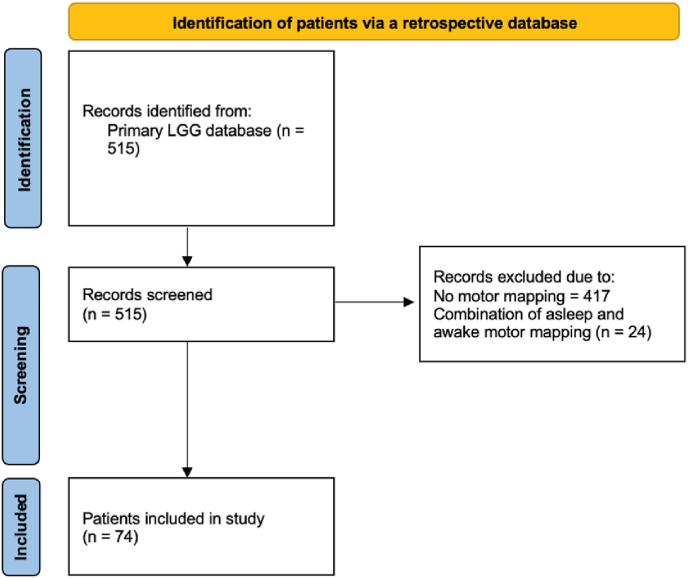
Flow chart of patient selection from main study.

The cohort included 74 patients from eight of the nine participating centers in the main study, with only one center that did not contribute any patients undergoing asleep motor mapping (Fig. 1).

Of these, 38 (51.4 %) were female, with a median age of 48 years (range 29–85). Seizures were the most common presenting symptom (57 patients, 77.0 %), and 55 (74.3 %) had a Karnofsky Performance Status of ≥90. A clinical deterioration was present in four patients before surgery. Twelve patients (16.2 %) presented with a preoperative motor deficit. *IDH* mutation was documented in 42 patients (56.8 %) and 1p19q codeletion in 32 (43.2 %). The median tumor volume was 43.2 ml. Detailed characteristics are shown in Table 1.

**Table 1:** Demographic, clinical, molecular, and radiological characteristics prior to surgery.

Variables	n (%)
**Female**	38 (51.4 %)
**Median age**/Q1-Q3	48 y/39.75–58.00
**Preop motor deficit**	9 (12.2 %)
**Preop seizure**	57 (77.0 %)
**Karnofsky score ≥ 90**	55 (74.3 %)
**Eloquence (UCSF score)**	61 (83.6 %)
**Tumor laterality** (right/left/bilateral)	45 (60.8 %)/28 (37.8 %)/1 (1.4)
**Contrast enhancement**	21 (28.4 %)
Patchy/diffuse/weak	11 (14.9 %)
Nodular	9 (12.2 %)
Ring-like	1 (1.4 %)
***IDH* mutated/not assessed**	42 (56.8 %)/27 (36.5 %)
***IDH* wildtype**	5 (6.8 %)
**1p19q codeletion/not assessed**	32 (43.2 %)/20 (27.0 %)
**Median pre-op tumor volume**/Q1-Q3	43.2 ml/16.0–79.3
**Gross total resection (0 ml residual)**	13 (17.6 %)
**Median post-op remnant**/Q1-Q3	7.81 ml/0.69–26.65
**fMRI pre-op**	41 (55.4 %)
**DTI pre-op**	55 (74.3 %)
**nTMS pre-op**	36 (48.6 %)

We explored characteristics of patients with permanent motor deficits. Among the 32 patients with any postoperative motor deficit, hemiparesis was the most common (16/32). Permanent deficits were more frequent in eloquent tumors (29.5 % vs. 8.3 %) and in lesions mainly located in the frontal lobe (38.1 %) compared to all other locations combined (9.4 %). Tumor volume did not differ meaningfully between those with (42.1 ml [17.7–74.8]) and without (44.3 ml [14.4–78.6]) permanent deficits.

**Fig. 2:**
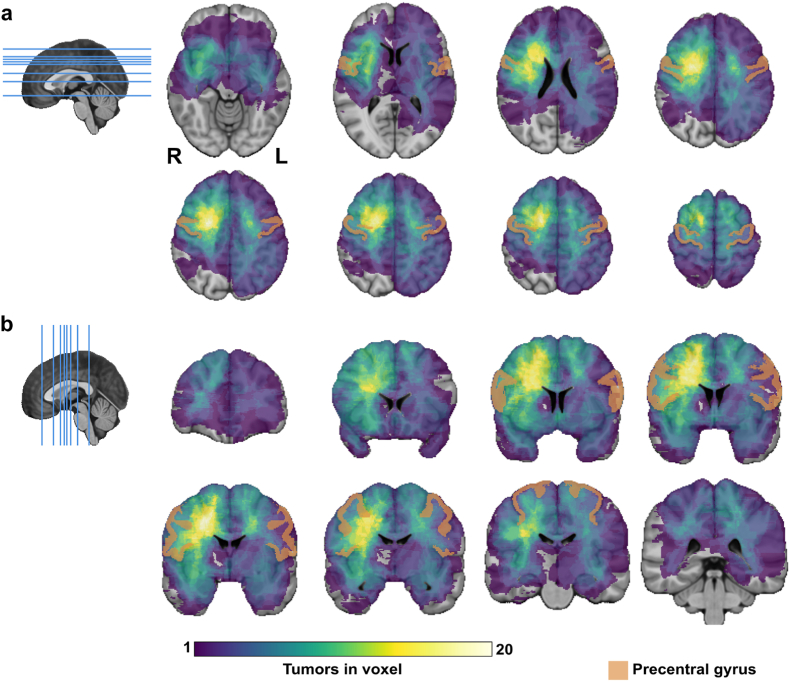
A heatmap visualizing the tumor location distribution of patients with LGG treated with asleep motor-mapping, a) heatmap in axial plane, b) coronal plane. Patients with available MRI for tumor segmentation were included, n = 71. The brightest color represents locations with the largest number of tumors in spatial overlap.

Tumors were predominantly located in the right hemisphere (45 patients, 60.8 %). Fig. 2 shows the heatmap of tumor locations.

### 3.2 Univariable and multivariable analyses of pre- and immediate postoperative factors

While the univariate analysis identified age, significant DWI changes, and pre-op motor deficits to be independent predictors of permanent post-op motor deficits, in the multivariable analysis only age and significant DWI changes remained significant (see Table 2).

**Table 2:** Comparison of significant factors in univariate and multivariate model.

Variables	Univariate	Multivariate
**Age**		
p-value (CIs)	0.043 (CI 1.01–2.28)	0.053
OR of 10 years	1.52	1.91 (CI 0.99–3.68)
**Significant DWI changes**		
p-value (CIs)	0.035 (CI 0.050–0.762)	0.012 (CI 0.03–0.64)
OR of 10 years	0.195	0.13
χ^2^	4.42	–
**Pre-op motor deficits**		
p-value (CIs)	0.009 (CI 1.76–36.33)	0.852 (CI 0.050–0.762)
OR of 10 years	8.00	1.26
χ^2^	6.74	–

### 3.3 Intraoperative characteristics and outcomes

Cortical and subcortical stimulation were used in 77 % and 81 % of patients, respectively. Continuous MEP monitoring was applied in 46 %. In 51 % of cases, surgery was stopped due to positive mapping findings (see Table 3, Table 4). Intraoperative seizures occurred in 11 % of patients.

**Table 3:** Mapping techniques.

Mapping technique	Count, n = 74 (%)
Cortical only	5 (6.8 %)
Subcortical only	7 (9.5 %)
Continuous MEPs only	3 (4.1 %)
Cortical + continuous MEPs	4 (5.4 %)
Cortical + Subcortical	26 (35.1 %)
Continuous MEPs + Subcortical	5 (6.8 %)
Cortical + continuous MEPs + Subcortical	22 (29.7 %)

**Table 4:** Intraoperative mapping and monitoring techniques.^a^.

Interoperative technique	Count, n = 74 (%)
Bipolar	25, (33.7 %)
Monopolar	51, (68.9 %)
ECOG	14, (18.9 %)
Relied on negative mapping	14, (18.9 %)
Positive sites identified in cortical stimulation	30/57, 24 missing, (52.6 %)
Positive sites identified in subcortical stimulation	31/60, 23 missing, (51.6 %)

a Includes also combination with multiple techniques

Postoperatively, 53 patients (71.6 %) experienced a new or worsened neurological deficit, whereas 31 (58.5 %) experienced motor deficits. Among the 32 patients with postoperative motor deficits (primary outcome including permanent pure-motor SMA but excluding motor–verbal SMA syndromes), hemiparesis was most common (n = 16, 50.0 %), followed by one-limb weakness (n = 12, 37.5 %) and isolated facial paresis (n = 4, 12.5 %) (see Table 5). Of these, hemiparesis was permanent in 12 cases, one-limb weakness in 3, and facial paresis in 1. SMA syndrome occurred in 15 patients (20.3 %), with 8 presenting motor-verbal symptoms and 7 pure motor symptoms. Three pure-motor SMA cases were (minor) permanent and thus included in the primary outcome. From 9 (12.2 %) patients suffering from language deficits, 7 experienced permanent with 2 being considered major deficits.

**Table 5:** Postoperative findings: motor deficits and DWI abnormalities.

Variable	n (%)
motor deficit post-surgery^a^ (permanent or transient)	32 (43.2 %)
Permanent deficit	19 (25.7 %)
Major deficit	5 (6.8 %)
Significant DWI changes	47 (63.5 %)
DWI changes >5 ml	5 (6.8 %)
Sector + rim shaped lesion types	7 (9.5 %)

a Hemiparesis, facial only, one limb, and SMA syndrome – pure motor only.

### 3.4 Associations with surgical parameters

Patients were grouped based on stimulation modality into “combined cortical and subcortical mapping” versus “single-method mapping” (either cortical or subcortical alone). No statistically significant differences in postoperative outcomes - including intraoperative seizures, new deficits, permanent deficits, or complication severity - were observed between patients receiving combined cortical and subcortical stimulation and those receiving only a single mapping modality (all p > 0.19) (Table 6).

**Table 6:** Comparison of key clinical outcomes across surgical modalities.

Group	All motor deficits	Permanent motor deficits	Intra-operative seizure	New-onset seizure	Ibañez grade ≥2
Cortical only	4/5 (80 %)	1/5 (20.0 %)	1/5 (20.0 %)	1/5 (20.0 %)	1/5 (20.0 %)
Subcortical only	1/7 (14.3 %)	1/7 (14.3 %)	0/7 (0.0 %)	0/7 (0.0 %)	1/7 (14.3 %)
Cortical + subcortical	12/26 (46.2 %)	5/26 (19.2 %)	4/26 (15.4 %)	1/26 (3.8 %)	4/26 (15.4 %)
Cortical + continuous MEPs + subcortical	11/22 (50.0 %)	8/22 (36.4 %)	3/22 (13.6 %)	4/22 (18.2 %)	8/22 (36.4 %)

### 3.5 Ischemic lesions

**Fig. 3:**
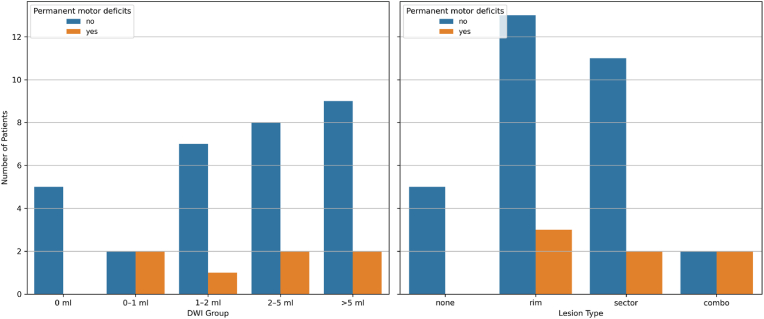
Occurrence of permanent motor deficits in different DWI groups and lesion types.

Data on lesion type and ischemic lesion volumes was available for 38 out of 74 patients (51.4 %) (Fig. 3). Among the 38 patients with valid postoperative DWI data, 86.8 % had measurable ischemic lesions (>0 ml).

The use of a transcortical approach was not significantly associated with ischemic volume (median 1.74 cm^3^ vs. 1.41 cm^3^; p = 0.4322). However, it was significantly associated with lesion type (χ^2^ = 12.72, p = 0.048). The association between transcortical access and lesion type was driven primarily by the association of transcortical access to rim-type lesions, which had the highest proportion of transcortical approaches (91 % vs 68 % in all other lesion types combined).

Sector lesions were seen in 9 transcortical (60.0 %) and 4 transsulcal/open-fissure approaches (26.7 %).

### 3.6 Seizure outcome and stimulation paradigm

Intraoperative seizure data were available for 67 (90.5 %), of whom 8 (11.9 %) experienced intraoperative seizures. In patients with available stimulation data, the median highest current was 8.5 mA [IQR: 5.75–13.5] in those with seizures (n = 6) and 10 mA [IQR: 6–20] in those without (n = 28). Given the small sample size, no statistical test was performed. Among patients who experienced intraoperative seizures (n = 8), 4 received monopolar stimulation (50 %) and 3 received both monopolar and bipolar (37.5 %). In the non-seizure group (n = 59), monopolar was used in 31 (52.5 %), bipolar in 6 (10.2 %), and both in 11 (18.6 %) patients.

No consistent pattern was observed across groups. There were no significant associations between intraoperative seizures and the use of seizure prophylaxis, stimulation mode (bipolar/monopolar), or the type of mapping technique applied (all p > 0.5). IDH-mutated tumors did not show substantially different rates of intraoperative seizures (6/37, 16.2 %) compared with IDH-wildtype (0/5, 0 %) and IDH not assessed (2/25, 8.0 %), and no statistically significant difference was observed (p = 0.70). Postoperative new-onset seizures or status epilepticus within 30 days were also more frequent among patients with intraoperative seizures (p = 0.023).

### 3.7 Other postoperative outcomes

Rehabilitation following surgery was recorded in 44 patients, and those who did receive rehabilitation most commonly required both inpatient and outpatient care (16 of 44). Postoperative complications within 30 days were reported in 17 patients, with superficial wound infections (n = 5) being the most common events (see Table 7). According to the Landriel Ibañez classification, most patients were graded as having no (n = 57) or minor complications (Grade I, n = 12).

**Table 7:** Complication within 30 days.

Complications	Count, n = 74 (%)
Superficial wound infection	5 (6.7 %)
Pneumonia	2 (2.7 %)
Epidural hematoma	2 (2.7 %)
Deep wound infection	1 (1.3 %)
Cavity hematoma	1 (1.3 %)
Pulmonary embolism	1 (1.3 %)
Hypokalemia	1 (1.3 %)
Transient decreased level of consciousness	1 (1.3 %)
Urinary retention	1 (1.3 %)
Diabetes insipidus and hypothalamic dysfunction	1 (1.3 %)
Bone flap removal	1 (1.3 %)
Atrial fibrillation	1 (1.3 %)

## 4 Discussion

In this multi-center cohort of 74 patients undergoing asleep motor mapping for LGG resection, we found that postoperative motor deficits were common, and 6.8 % of patients suffered major permanent motor deficits postoperatively. Significant DWI changes postoperatively and age were persistently independent predictors for new/worsened motor deficits that were permanent. Intraoperative seizures, as seen in 11.9 % were associated with risk of postoperative epilepsy.

### 4.1 Preoperative motor deficits

In LGG, seizures and motor deficits are among the most commonly reported symptoms at presentation (Deacu et al., 2022; Kivioja et al., 2023). In line with our findings, a recent meta-analysis from Conway et al. (2025) with over 2600 patients with brain tumors support our findings of pre-operative motor deficits being a strong predictor for post-operative dysfunction (Conway et al., 2025). Adding to this finding is Zetterling et al. (2020) showing that patients with pre-existing deficits are more likely to experience non-reversible deterioration (Zetterling et al., 2020). While our cohort focused exclusively on asleep motor mapping, previous studies comparing awake and asleep surgery have highlighted that differences in postoperative outcomes may be influenced by patient selection rather than mapping technique itself. In line with this, a recent review concluded that the rates of transient and permanent postoperative neurological deficits are comparable between awake (10.8 %) and asleep cortical mapping (12.7 %) approaches, supporting the use of both techniques to minimize perioperative morbidity, with comparable rates of permanent deficits observed when adjusted for individual clinical factors (Kurian et al., 2022).

### 4.2 Mapping and monitoring techniques

In our study, we found no statistically significant differences in postoperative motor outcomes between the mapping and monitoring techniques employed. This may partly reflect limited sample sizes per subgroup, but also suggests that other factors - such as tumor location, preoperative deficits, and intraoperative anatomical constraints (e.g. proximity to motor pathways) - may have a greater impact on outcome. The heatmap of tumor locations in our study showed that the majority of the tumors were located posteriorly in the subcortical right frontal lobe. The restriction to asleep motor-mapping likely underrepresents left-hemisphere lesions, which may be preferentially managed awake for language risks.

A recent study introduced a validated motor mapping score to guide the choice between awake and asleep procedures based on preoperative deficits, tumor volume, prior treatment, and praxis network involvement, supporting individualized intraoperative strategies over a one-size-fits-all approach (Rossi et al., 2022). Previous studies have reported associations between certain stimulation modalities, such as monopolar subcortical mapping, and postoperative deficits (Staub-Bartelt et al., 2023), but such findings may be confounded by underlying case complexity. Moreover, lower subcortical stimulation thresholds during continuous short-train monopolar mapping (i.e., low mA indicating proximity to motor pathways) have been linked to transient postoperative motor deficits that resolve, underscoring the interplay between mapping readouts, resection limits, and outcomes (Lutz et al., 2023).

Our finding that significant postoperative DWI changes were independently associated with permanent motor deficits further highlights that factors beyond mapping and monitoring often contribute to functional decline. Krieg et al. (2012) (Krieg et al., 2012) similarly reported postoperative deficits despite stable MEPs, often due to secondary ischemia, hemorrhage, or SMA involvement. Strand et al. (2022) also found peritumoral infarctions in 38 % of glioma resections, which were not reliably predicted by intraoperative monitoring, emphasizing the role of subtle surgical and anatomical variables (Strand et al., 2021). These results are echoed in high-grade glioma cohorts, where postoperative infarcts correlate with early motor deficits and reduced postoperative KPS (Berger et al., 2022), suggesting that ischemic injury has functional consequences across glioma grades.

### 4.3 Intraoperative seizures and post-operative seizure risk

Intraoperative seizures were associated with a higher rate of early postoperative seizures and diffusion abnormalities, suggesting increased seizure susceptibility in some patients or aggravation of postoperative seizure risk following an intraoperative seizure. Cordella et al. (2013) reported intraoperative seizures as a non-negligible complication during motor mapping that can interrupt mapping. In their cohort, seizure occurrence was not associated with higher stimulation intensities (Cordella et al., 2013). Our observed seizure rate (11.9 %) is in line with other reports on intraoperative stimulation-related seizures in both awake and general anesthesia settings (ca. 10.5 %–13.7 %) (Morsy et al., 2021; Fountas et al., 2022; Youshani et al., 2024). Importantly, intraoperative seizure management is not standardized across centers worldwide, with considerable variability in the use of irrigation, antiepileptic medication, and anesthetic suppression during stimulation. Such heterogeneity may influence reported seizure rates across studies and reflects the diversity of real-world practice (Jasper et al., 2025).

As highlighted by our findings, cortical and subcortical stimulation at higher intensities did not significantly predict seizure occurrence, reinforcing the multifactorial nature of intraoperative excitability (Morsy et al., 2021). While intraoperative events appear to increase early postoperative risk within this continuum, they may not substantially affect long-term seizure control (Pauletto et al., 2025).

### 4.4 Perioperative neurophysiology and future perspectives on motor recovery

Alterations in interhemispheric cortical excitability have been associated with postoperative motor vulnerability and reduced recovery potential in glioma patients, highlighting a possible role for neurophysiology-informed rehabilitation strategies (Rosenstock et al., 2017, 2022). Therapeutic approaches targeting these mechanisms show promise, as contralesional low-frequency repetitive TMS (rTMS) may facilitate early motor recovery after glioma surgery, particularly in patients with ischemic injury (Rosenstock et al., 2025; Engelhardt et al., 2024). Since a non-negligible proportion of patients experienced significant postoperative motor deficits, there is also a need for effective rehabilitation. Novel rehabilitation strategies like rTMS may represent one way of improving motor recovery in future patients.

### 4.5 Limitations

This study draws on data from eight centers across with population-based referral, reflecting diverse real-world surgical practices in asleep motor mapping rather than necessarily “best practice”. Despite the limited sample size, the dataset is rich, capturing detailed variables from the preoperative, intraoperative, and postoperative phases, including advanced imaging, stimulation parameters, and clinical outcomes. Image analyses of tumor volumes and DWI changes were centralized. The structured and comprehensive approach enables a nuanced analysis of factors influencing motor and seizure outcomes in LGG surgery.

A key limitation of this study is the relatively small sample size which may limit statistical power and the ability to detect subtle associations. Nevertheless, the limited sample size reflects the low incidence of newly diagnosed WHO grade 2 low-grade gliomas treated. Many LGG are located more anteriorly (Zetterling and Latini, 2024) where motor mapping may be considered unnecessary. Also, as seen with the right predominance of tumors in our cohort, patients with left-sided tumors were presumably more often treated under awake conditions. Another remark is also that molecular testing for IDH was incomplete for a subset (IDH not assessed) given the study period that predated routine IDH testing. Exploratory comparisons, including percentage of intraoperative seizures, showed no meaningful differences.

Additionally, the dataset provided only a single overall ‘highest current used on direct stimulation’ value per case. Separate maximal currents for cortical and subcortical stimulation were not recorded, which prevented a site-specific analysis of stimulation thresholds and seizure risk.

The binary grouping of certain variables such as DWI volume and postoperative deficits, although necessary for analysis, may have oversimplified the clinical complexity. For instance, we recoded postoperative permanent deficits, such as permanent hemiparesis, one limb paresis, or permanent facial only deficit, into one permanent motor deficit category. In addition, although we recorded the highest stimulation current, we did not capture the minimum current (threshold, mA) eliciting a positive response. Therefore, we cannot relate threshold-based safety margins to resection stop decisions. Further, the data is from surgical procedures performed 2012–2017 and may not fully capture the current situation. The retrospective data capture may also lack granularity for complex variables and introduce bias typical for retrospective studies. Lastly, we recorded motor outcomes only and did not capture cognitive deficits, and because the cohort included asleep-mapped cases only, we did not assess postoperative deficits in awake surgeries, limiting generalizability.

## 5 Conclusions

Our findings demonstrate the case selection for asleep motor mapping in LGG and highlight the critical role of preoperative deficits and postoperative DWI changes in shaping functional outcomes after surgery. While mapping and monitoring modalities varied, no single approach emerged as superior. Future studies should focus on establishing a “best practice”, refining intraoperative risk prediction - integrating neurophysiological, radiological, and clinical markers - to guide surgical planning and improve outcomes in low-grade glioma surgery.

## Funding

The study was financed by grants from the Swedish state under the agreement between the Swedish government and the county councils, the Avtal om Läkarutbildning och forskning (ALF)-agreement: ALFGBG-965622 and ALFGBG-1006089 (10.13039/100008329ASJ).

## Declaration of competing interest

The authors declare the following financial interests/personal relationships which may be considered as potential competing interests: F.L. received an honorarium from Servier AG, which is not related to the manuscript. All other authors declare no competing financial interest/personal relationships that might have influenced the process of this manuscript.
