# Supplementary material for: Asleep motor mapping in resected low-grade gliomas -a population based multicenter study
Source: Brain Spine. 2025 Dec 23;6:105918. doi: 10.1016/j.bas.2025.105918 (PMC12796598; doi:10.1016/j.bas.2025.105918)
Supplement: Multimedia component 1 [file mmc1.docx]

**Supplementary material**

Suppl. Table 1. Center-level utilization of asleep motor mapping of large STAR cohort

| **Patient center** | **Asleep mapping n (%)** |
| --- | --- |
| 1 | 16/65 (24.6%) |
| 2 | 15/110 (13.6%) |
| 3 | 14/75 (18.7%) |
| 4 | 9/30 (30.0%) |
| 5 | 8/73 (11.0%) |
| 6 | 6/36 (16.7%) |
| 7 | 3/55 (5.5%) |
| 8 | 3/52 (5.8%) |
| 9 | 0/19 (0.0%) |

Suppl. Table 2. MRC Muscle Power Scale

Minor

Major

| **Score** | **Description** |
| --- | --- |
| 0 | No contraction |
| 1 | Flicker or trace of contraction |
| 2 | Active movement, with gravity eliminated |
| 3 | Active movement against gravity |
| 4 | Active movement against gravity and resistance |
| 5 | Normal power |
